# Mode of delivery and offspring atopic dermatitis in a Swedish nationwide study

**DOI:** 10.1111/pai.13904

**Published:** 2023-01-11

**Authors:** Mwenya Mubanga, Cecilia Lundholm, Elin S. Rohlin, Gustaf Rejnö, Bronwyn K. Brew, Catarina Almqvist

**Affiliations:** ^1^ Department of Medical Epidemiology and Biostatistics Karolinska Institutet Stockholm Sweden; ^2^ Obstetrics and Gynaecology Unit Stockholm Sweden; ^3^ National Perinatal Epidemiology and Statistics Unit, Centre for Big Data Research in Health and School of Clinical Medicine University of New South Wales Sydney Australia; ^4^ Pediatric Allergy and Pulmonology Unit at Astrid Lindgren Children's Hospital Karolinska University Hospital Stockholm Sweden

**Keywords:** atopic dermatitis, caesarean section, eczema, mode of delivery, sibling

## Abstract

**Background:**

Atopic dermatitis is a common chronic childhood disease associated with significant morbidity and healthcare costs. There is a known association between caesarean section and asthma, but the relationship between caesarean section and offspring atopic dermatitis remains uncertain.

**Methods:**

We conducted a register‐based nationwide cohort study including children born in Sweden between January 2006 and December 2018. Data on health and socioeconomic variables were extracted from the national registers for children aged ≤5 years. Time‐to‐event analyses were used to calculate hazard ratios (HR) with 95% confidence intervals (CI) adjusting for confounders and familial factors.

**Results:**

1,399,406 children were included (6,029,542 person‐years at risk). Atopic dermatitis was observed in 17.2% of the 1,150,896 children born by vaginal delivery and 18.3% of the 248,510 born by caesarean section. The mean age of onset of atopic dermatitis was 2.72 years (SD 1.8). Birth by caesarean section was associated with a higher risk of atopic dermatitis (adj‐HR 1.12, 95% CI: 1.10–1.14). A higher risk of atopic dermatitis was found in children born by instrumental vaginal delivery (adj‐HR 1.10, 1.07–1.13); emergency caesarean section (adj‐HR 1.12, 1.10–1.15), and elective caesarean section (adj‐HR 1.13, 1.10–1.16) than uncomplicated vaginal delivery in children <1 year of age. Similar hazards were observed in those ≥1 year of age. In sibling control analysis, greater risks remained in children aged <1 year but not in age ≥1 year.

**Conclusions:**

In our study population, it was observed that children born by caesarean section or instrumental vaginal delivery were at higher risk of early childhood atopic dermatitis. Although familial confounding attenuates the risk in children aged ≥1 year, this was not observed in the first year of life.

## Graphical abstract

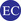

**Key Message**

Our study found that there is an increased risk for atopic dermatitis in children aged ≤5 years born by caesarean section.

Birth by instrumental vaginal delivery, emergency, and elective caesarean delivery was associated with atopic dermatitis.

In the sibling control analysis, birth by instrumental vaginal delivery, emergency, and elective caesarean delivery was associated with atopic dermatitis in children aged <1 year indicating some familial confounding.

## 1 BACKGROUND

Allergic diseases and atopic conditions such as atopic dermatitis have risen globally, and yet the reason for this increase is not fully elucidated.^1^ Atopic dermatitis is the most prevalent chronic noncommunicable disease of the skin globally.^2^ It often presents as a relapsing condition characterized by pruritus, abnormally dry skin, and eczematous lesions.^3^ The disease displays a high heterogeneity in its natural course and individual trajectories are unpredictable; however, it may have systemic effects that increase the likelihood of other comorbidities. Apart from its association with other atopic conditions such as food allergy and asthma,^4^ children and adults with eczema are also more likely to experience more frequent bacterial and viral infections.^5^, ^6^ Other comorbidities include overweight/obesity^7^ and also psychiatric conditions such as depression, anxiety, and autism.^8^ The condition is often mild, but in some children and adults may be debilitating, require constant medication and significantly impair the quality of life. Further, Sweden ranks as having one of the highest disability‐adjusted life‐years for atopic dermatitis globally making it an important place in which to investigate the condition.^9^

It has been suggested that the increased atopic dermatitis risk may be partially explained by the increase in caesarean sections across the globe.^10^, ^11^, ^12^ In the last decade, caesarean sections have risen globally from 9% to 21%, with Europe reporting rates of circa 25.7% (23.4–28.0) in 2018.^13^ Caesarean sections have been linked with decreased gut microbial diversity and a reduced ability of the infant body to develop adequate immune response mechanisms.^14^ Whilst the indications for births by caesarean section are multifactorial, the mode of delivery may have clinical outcomes for children, including atopic dermatitis.^15^

Previous studies have examined the association between birth by caesarean section and atopic dermatitis with inconsistent results. In various studies, including a prospective Australian birth cohort study (*n* = 10,383),^16^ the United States National Surveys of Children's Health birth cohort study (*n* = 249,585),^17^ and the British West Midlands birth cohort study (*n* = 24,690)^18^ there was no association found.^16^, ^17^, ^18^ By contrast, a positive association between caesarean section and atopic dermatitis was observed in both a study conducted in Korean adolescents (*n* = 1302),^19^ and in Ecuadorian children (*n* = 400).^20^ As caesarean section may be associated with asthma,^11^, ^21^ and asthma and atopic dermatitis are genetically and phenotypically linked, the inconsistent results observed in different studies need to be explored further.

Some studies do not account for the temporal risk of early onset atopic dermatitis or the different types of methods of delivery. Birth by caesarean section has been associated with an altered gut microbiome,^22^ but the differences between elective and emergency caesarean sections, or those between uncomplicated and complicated vaginal deliveries are not regularly accounted for. The use of different definitions of atopic dermatitis including differences based on physician or parent reporting, and an inability to adjust for different familial and socioeconomic confounders, including those shared by siblings, may also limit the generalizability of findings.

The aim of this nationwide register‐based cohort study of Swedish mother and child pairs was to investigate the association between mode of delivery and atopic dermatitis in children aged ≤5 years. We additionally conducted a sibling analysis to adjust for unmeasured familial factors, thus further assessing causality.^23^

## 2 METHODS

### 2.1 Study population

This register‐based cohort study included all singleton children born in Sweden between January 2006 and December 2018 (*n* = 1,400,713) who were identified in the Medical Birth Register.^24^ This register includes ≥98% of all births in Sweden and reports on antenatal events, deliveries, and birth characteristics. By using a unique personal identity number,^25^ information about mother and child was linked to other registers for health and socioeconomic factors. These include the longitudinal integrated database for health insurance and labour market studies (LISA)^26^ for information about educational level; the Medical Birth Register for information on siblings^27^; the National Patient Register^28^ for disease identification and the Swedish Prescribed Drug Register^29^ for information on prescribed drugs. The National Patient Register covers all inpatient hospital diagnoses since 1987 and includes specialist outpatient diagnoses since 2001.^28^ The Swedish Prescribed Drug Register covers all dispensed prescribed drugs in Sweden from July 2005.^29^ We excluded children who had missing information on the mode of delivery (*n* = 1286) and children with missing information on maternal pregnancy (*n* = 21), Figure S1. Siblings were identified by shared maternity in the Medical Birth Register, and these were included in the sibling analysis (*n* = 853,884), of which 320,692 were discordant.

### 2.2 Outcome

Atopic dermatitis was defined using a clinical algorithm established by Henriksen et al.^30^ This is based on either a diagnosis of atopic dermatitis in the National Patient Register or the dispense of drugs used in the treatment of atopic dermatitis as identified from the Swedish Prescribed Drug Register. A full description of the criteria is included in the Appendix S1.

### 2.3 Exposure

Mode of delivery was extracted from the Medical Birth Register.^24^ It was first defined as birth by vaginal delivery or caesarean section. Using information from the Medical Birth Register on the type of delivery, spontaneous onset of labour, caesarean section (elective or emergency caesarean section prior to the onset of labour, or emergency caesarean section after the onset of labour), and vaginal instrumental delivery were extracted. Based on these, the mode of delivery was categorized as: (1) vaginal, uncomplicated delivery, (2) vaginal, instrumental delivery, (3) emergency caesarean section (prior to or after start of labour) and (4) elective caesarean (before start of labour).

### 2.4 Other variables

Potential confounders were identified based on prior knowledge and a directed acyclic graph, Figure S2. Child‐related factors extracted from the Medical Birth Register included: sex (male/female), birth weight (continuous), birth year (discrete), and gestational age at birth (weeks). Maternal factors included: maternal age (continuous) and maternal parity. Maternal history of asthma was based on a validated algorithm using medication information from the Swedish Prescribed Drug Register and diagnosis information from the National Patient Register.^31^ The LISA was used for information on the maternal level of education; compulsory (≤9 years), secondary (10–11 years), or tertiary (≥12 years) at the time of the child's birth.

### 2.5 Statistical analyses

Time‐to‐event analysis using Cox proportional hazards regression models was performed with attained age as the analysis time scale to assess the association between mode of delivery and atopic dermatitis. The cluster robust sandwich estimator for standard errors was used to account for clustering of observations within families (siblings). The proportional hazards assumption was tested based on Schoenfeld residuals and found to be violated. We therefore allowed for time‐varying effects, where follow‐up time was divided into two intervals: age <1 year and age ≥1 year. The follow‐up was restricted to age 5 as ≥80% of the childhood onset of atopic dermatitis occurs before age 5.^32^ Observations were censored at age 5, emigration, death, or at the end of the study on December 2021 (whichever came first). Models were presented as (i) age‐adjusted (as analysis time scale), (ii) age‐ and sex‐adjusted, and (iii) adjusted for age, sex, birthweight, gestational age, maternal age, parity, maternal history of asthma, and maternal level of education.

Further, we conducted a sibling control analysis to account for unmeasured familial confounders, both environmental and genetic, shared by siblings using stratified Cox proportional hazards regression. Siblings were identified by shared maternity in the Medical Birth Register and restricted to siblings with the same mother. Siblings discordant for exposure and outcome were informative for the estimation of the hazard ratios for the association of interest, although all siblings were included in the analyses as they would contribute to the estimation of covariate coefficients. The sibling models were adjusted for covariates not shared by siblings, that is, child's age, sex, gestational age, birthweight, parity, and maternal age. All results from Cox proportional hazards regression were reported as hazard ratios (HR) and 95% confidence intervals (CI). Analysis was performed using STATA 16.^33^

### 2.6 Ethics statement

All data were pseudonymized prior to analysis. Study participants were not involved in the planning or writing of this research project. Ethical approval was obtained for this project from the Swedish Ethical Review Authority.

## 3 RESULTS

The study included 1,399,406 children after excluding 1307 children with missing information on the mode of delivery or with missing information on the mother's identity, Figure S1. The mean age of the children included in the study was 4.2 years (SD 1.3) at the end of follow‐up, and 719,604 (51.4%) were boys. More children were born by vaginal delivery than caesarean section (82.2% and 17.8%, respectively), but more children were born at or before 34 full weeks of gestation in the group delivered by caesarean section compared with vaginal delivery (7.4% and 1.3%, respectively). Mothers with a vaginal delivery were on average younger than those who delivered by caesarean section (30.1 years (SD 5.1) vs. 31.8 years (SD 5.3), respectively). Other baseline characteristics are shown in Table 1. The population baseline characteristics when vaginal delivery was subdivided into uncomplicated and instrumental (forceps or vacuum) vaginal delivery and caesarean section into emergency and elective caesarean sections are shown in Table S1.

**TABLE 1:** Baseline characteristics of mother and child pairs.

	Total population	Vaginal delivery	Caesarean section
*N* (%)	1,399,406 (100)	1,150,896 (82.2)	248,510 (17.8)
Child Characteristics			
Child's age at end of follow‐up in years (mean, SD)	4.3 (1.3)	4.3 (1.3)	4.3 (1.4)
Sex			
Male	719,604 (51.4)	587,550 (51.1)	132,054 (53.1)
Gestational age (weeks)			
≤31	12,318 (0.9)	4354 (0.4)	7.964 (3.2)
32–34	20,584 (1.5)	10,066 (0.9)	10,518 (4.2)
35–36	47,026 (3.4)	31,703 (2.8)	15,323 (6.2)
37–38	265,423 (19.0)	181,844 (16.8)	83,579 (33.6)
39–40	710,282 (50.7)	624,402 (54.2)	85,880 (34.6)
≥41	343,474 (24.5)	298,287 (25.9)	45,187 (18.2)
Missing	299 (<0.0)	240 (<0.0)	59 (<0.0)
Birthweight (grams)			
Mean (SD)	3511 (583)	3543 (528)	3367 (772)
Maternal Characteristics			
Maternal age (years)			
Mean (SD)	30.4 (5.2)	30.1 (5.1)	31.8 (5.3)
Parity			
1	605,044 (43.2)	489,225 (42.5)	115,819 (46.6)
2	516,968 (36.9)	432,701 (37.6)	84,267 (33.9)
3	192,460 (13.8)	158,878 (13.8)	33,582 (13.5)
≥4	84,934 (6.1)	70,092 (6.1)	14,842 (6.0)
Level of education			
Compulsory (≤9 years)	127,826 (9.1)	105,982 (9.2)	21,844 (8.8)
Secondary (10–11 years)	494,097 (35.3)	406,201 (35.3)	87,896 (35.4)
Tertiary (≥12)	766,126 (54.8)	629,164 (54.7)	136,962 (55.1)
Missing	11,357 (0.8)	9549 (0.8)	1808 (0.7)

### 3.1 Association of mode of delivery with atopic dermatitis

In this population of 1,399,406 children, 243,293 (17.4%) developed atopic dermatitis at a mean age of 2.72 years (SD 1.8). In total children, were followed up for 6,029,542 person‐years. For children aged less than 1 year, birth by caesarean section was associated with a higher risk of atopic dermatitis in the age‐, age and sex and fully‐adjusted models (HR 1.11, 95% CI: 1.09–1.11; HR 1.10, 95% CI: 1.08–1.12 and adj‐HR 1.12, 95% CI: 1.10–1.14, respectively) when compared to vaginal delivery. In children aged ≥1 year, the HRs for atopic dermatitis were also increased although lower than <1‐year estimates in the age‐, age and sex‐ and fully‐adjusted models (HR 1.06, 95% CI: 1.04–1.07; HR 1.06, 95% CI: 1.04–1.07 and adj‐HR 1.05, 95% CI: 1.04–1.07, respectively) Table 2.

**TABLE 2:** Associations between mode of delivery (vaginal delivery vs caesarean section) and atopic dermatitis.

	Atopic dermatitis	Population in full analysis	Model 1 HR (95% CI)	Model 2 HR (95% CI)	Model 3 HR (95% CI)
Age <1 year					
Vaginal delivery	68,443	1,150,896	Ref	Ref	Ref
Caesarean section	16,269	248,510	1.11 (1.09–1.13)	1.10 (1.08–1.12)	1.12 (1.10–1.14)
Age ≥1 year					
Vaginal delivery	207,647	1,080,117	Ref	Ref	Ref
Caesarean section	47,065	230,593	1.06 (1.04–1.07)	1.06 (1.04–1.07)	1.05 (1.04–1.07)

*Note*: Model 1: Crude model with age as a time scale. Model 2: Adjusted for child's age and sex. Model 3: Adjusted for child age, sex, birth weight, gestational age, birth year, maternal age, parity, maternal history of asthma, and level of education.

In the analysis by mode of delivery subgroup, for those aged <1 year, when compared to uncomplicated vaginal delivery, those born by instrumental vaginal delivery were at higher risk of atopic dermatitis (adj‐HR 1.10, 95% CI: 1.07–1.13); as were those born by emergency caesarean section and elective caesarean section (adj‐HR 1.12, 95% CI: 1.10–1.15 and adj‐HR 1.13, 95% CI: 1.10–1.16, respectively), fully‐adjusted model, Table 3. Similarly, in those aged ≥1 year, when compared to vaginal delivery, those born by instrumental delivery, emergency caesarean section, and elective caesarean section showed an increased risk of atopic dermatitis although lower compared with <1‐year estimates (adj‐HR 1.04, 95% CI: 1.02–1.06, adj‐HR 1.05, 95% CI: 1.03–1.07 and adj‐HR 1.06, 95% CI: 1.04–1.09), respectively Table 3. An illustration of the survival estimates is shown in Figure S3.

**TABLE 3:** Associations between mode of delivery (vaginal delivery vs. instrumental delivery, emergency, and caesarean section) and atopic dermatitis.

	Atopic dermatitis	Population in full analysis	Model 1 HR (95% CI)	Model 2 HR (95% CI)	Model 3 HR (95% CI)
Age <1 year					
Uncomplicated vaginal delivery	62,428	1,056,323	Ref	Ref	Ref
Instrumental vaginal delivery	6015	94,573	1.08 (1.05–1.11)	1.06 (1.03–1.09)	1.10 (1.07–1.13)
Emergency caesarean section	9210	140,806	1.12 (1.09–1.14)	1.10 (1.08–1.13)	1.12 (1.10–1.15)
Elective caesarean section	7059	107,704	1.11 (1.09–1.14)	1.11 (1.08–1.14)	1.13 (1.10–1.16)
Age ≥1 year					
Uncomplicated vaginal delivery	189,036	991,752	Ref	Ref	Ref
Instrumental vaginal delivery	18,612	88,365	1.03 (1.01–1.05)	1.03 (1.01–1.05)	1.04 (1.02–1.06)
Emergency caesarean section	26,814	130,264	1.07 (1.05–1.09)	1.07 (1.05–1.09)	1.05 (1.03–1.07)
Elective caesarean section	20,251	100,329	1.05 (1.03–1.07)	1.05 (1.03–1.07)	1.06 (1.04–1.09)

*Note*: Model 1: Crude model with age as a time scale. Model 2: Adjusted for child's age and sex. Model 3: Adjusted for child's age, sex, birth weight, gestational age, birth year, maternal age, parity, maternal history of asthma, and level of education.

### 3.2 Sibling analysis

In sibling analyses, compared with vaginal delivery, in children aged <1 year, birth by caesarean section was associated with atopic dermatitis (adj‐HR 1.06, 95% CI: 1.01–1.11). However, in those aged ≥1 year, there was no elevated risk observed (adj‐HR 0.96, 95% CI: 0.93–1.00), Table 4.

**TABLE 4:** Sibling analysis for the associations between mode of delivery and atopic dermatitis. Representing 853,884 pairs of whom 320,692 were discordant.

	Atopic dermatitis	Model 1 HR (95% CI)	Model 2 HR (95% CI)	Model 3 HR (95% CI)
Age <1 year				
Vaginal delivery	36,159	Ref	Ref	Ref
Caesarean section	9311	1.05 (1.00–1.10)	1.03 (0.98–1.08)	1.06 (1.01–1.11)
Age ≥1 year				
Vaginal delivery	39,839	Ref	Ref	Ref
Caesarean section	9519	0.99 (0.96–1.03)	0.99 (0.96–1.03)	0.96 (0.93–1.00)
Age <1 year				
Uncomplicated vaginal delivery	32,515	Ref	Ref	Ref
Instrumental vaginal delivery	3644	0.89 (0.85–0.94)	0.87 (0.83–0.92)	1.10 (1.05–1.16)
Emergency caesarean section	5538	0.99 (0.94–1.04)	0.96 (0.91–1.01)	1.11 (1.05–1.18)
Elective caesarean section	3773	1.08 (1.02–1.15)	1.08 (1.01–1.14)	1.04 (0.97–1.10)
Age ≥1 year				
Uncomplicated vaginal delivery	36,223	Ref	Ref	Ref
Instrumental vaginal delivery	3616	0.89 (0.86–0.93)	0.89 (0.86–0.92)	0.98 (0.95–1.02)
Emergency caesarean section	5550	0.94 (0.90–0.98)	0.93 (0.90–0.97)	0.96 (0.92–1.00)
Elective caesarean section	3969	1.02 (0.98–1.07)	1.02 (0.98–1.07)	0.96 (0.92–1.01)

*Note*: Model 1: Crude model with age as a time scale. Model 2: Adjusted for child's age and sex. Model 3: Adjusted for child's age, sex, birth weight, gestational age, parity.

Similarly, in the mode of delivery subgroup analysis, when compared to uncomplicated vaginal delivery, in those aged <1 year, there was an increased risk of atopic dermatitis observed in children born by instrumental vaginal delivery (adj‐HR 1.10, 95% CI: 1.05–1.16) and emergency caesarean section (adj‐HR 1.11, 95% CI: 1.05–1.18) but not for those with elective caesarean section (adj‐HR 1.04, 95% CI: 0.97–1.10), Table 4.

In those aged ≥1 year, compared with uncomplicated vaginal delivery, there was no increased risk of atopic dermatitis in children born by instrumental vaginal delivery (adj‐HR 0.98, 95% CI: 0.95–1.02), emergency caesarean section (adj‐HR 0.96, 95% CI: 0.92–1.00) or by elective caesarean section (adj‐HR 0.96, 95% CI: 0.92–1.01), Table 4.

## 4 DISCUSSION

In this prospective population‐based study investigating the association between mode of delivery and atopic dermatitis, a positive association between birth by caesarean section and the risk of atopic dermatitis was observed in the whole population regardless of whether the period of observation was before 1 year of age or between one and 5 years of age. In the analysis that further subdivided the mode of delivery, the risk of atopic dermatitis was higher in children born by instrumental delivery, emergency, and elective caesarean section compared with uncomplicated vaginal delivery regardless of the age of observation. Similar risks for atopic dermatitis were observed for children exposed to emergency caesarean section and those born by elective caesarean section. In the sibling analysis controlling for familial and environmental factors, the increased risk of atopic dermatitis was still observed in those born by caesarean section compared with vaginal delivery in children aged less than 1 year and completely attenuated for one to 5 years of age. The sibling analysis results support a possible causal association before age 1 year may be confounded by the indication for the mode of delivery; however, the association after age one can be attributed to shared environmental and genetic factors rather than being causal in nature.

Overall, our study found that 17% of children in Sweden aged 0–5 years had atopic dermatitis between 2006 and 2021. This is an increase over our previous observation of 11.4% in overlapping similar populations in Sweden and highlights the public health importance of this medical condition for children and the need for research into aetiological pathways.^34^ This increase may be due to a rise in the prevalence of eczema in the Swedish pediatric population or that there has been an increase in diagnoses particularly in specialist care. In 2016, Sweden also introduced free medication for children, which may have increased parental health‐seeking behavior.

A recent international epidemiological study that included pediatric findings from South America, Europe, the Middle East, and the United States, showed that diagnosed atopic dermatitis ranged from 9.8% to 20.1% in children aged 6 months to 6 years; however, the authors reported that there was no clear trend in prevalence by age, sex or geographical location.^2^ The prevalence of Sweden is similar to other countries in the region.^2^

### 4.1 Comparison to other studies

To the best of our knowledge, only two previous studies have shown an association between mode of delivery and atopic dermatitis.^19^, ^35^ In the Korean study by Yu et al, (*n* = 1302; OR 1.80, 95% CI: 1.14–2.85)^19^ there was higher odds of atopic dermatitis, but not asthma, in an adolescent population. In addition to variables we adjusted for, the researchers also adjusted for serum 25‐hydroxyvitamin D level, fat intake, and breastfeeding. In the Ecuadorian study by Gorris et al (*n* = 400; OR 2.65, 95% CI: 1.06–6.61),^20^ the study participants were aged 3–12 years and also adjusted for breastfeeding, daycare attendance, paternal allergy status, parental smoking, and ethnicity. We did not adjust for diet, breastfeeding, or daycare attendance because we considered them mediators of the relationship between the mode of delivery and atopic dermatitis association rather than confounding structures. Although we could not account for ethnicity directly in our study, we adjusted for the maternal country of birth as a proxy and observed no difference in the overall estimates (results not shown). Further, by conducting a nationwide study, we were also able to include children of different ethnicities. There is a predominance of studies on individuals of European ancestry, and few nationwide studies that account for ethnic diversity. Atopic dermatitis, however, is more common and severe *in non‐Caucasian than Caucasian* children.^36^ Skin biopsy specimens from Asian children with atopic dermatitis express significantly higher levels of T helper Th17‐ and T_h_22‐related cytokines (especially interleukin (IL)‐17A, IL‐19, and IL‐22) and IL‐17/IL‐22 than found in skin biopsy specimens from European American children.^37^ These findings account for the increased psoriasiform changes in Asian patients with atopic dermatitis because IL‐19 and IL‐22 are known to induce epidermal hyperplasia and parakeratosis. Immune activation pathways in African American patients with atopic dermatitis are yet to be described.^37^

Our results are different from two large studies conducted on the mode of delivery and offspring atopic dermatitis. In the United States National Surveys of Children's Health birth cohort study (*n* = 249,585),^17^ no association was observed. This may be due to limitations in the ability to capture the outcome. Although the birth cohort covered the period of 1990 to 2011, eczema measures were only captured in 2003 and 2007, and the population included in the study for the analysis on eczema reduced to 150,000.^17^ In the British West Midlands by McKeever et al (24,690)^18^ the prevalence of eczema was very high (>30%), which may account for the differences from the current study.

It is postulated that since caesarean delivery deprives the newborn exposure to maternal vaginal and fecal flora, this results in an altered microbiome in the child and may predispose to disease in later life.^14^ The gut microbiome plays a pivotal role in the balance between the inflammatory Th‐cells and immune tolerance under healthy conditions.^38^ In the pooled European cohort study by Adlerberth et al., (*n* = 324)^39^ birth by caesarean section was associated with delayed acquisition of species from the families *Bifidobacteriaceae* and *Bacteroidaceae* but instead associated with compensatory colonization by species from the families *Enterobacteriaceae* and *Clostridiaceae*.^39^ Delayed acquisition of the *Bifidobacteriaceae* and *Bacteroidaceae* at 1 month is associated with an increased risk of atopic dermatitis in later life.^40^ The findings were corroborated in the studies by Reyman et al.^41^ and Jakobsson et al.^14^; the latter also found that children born by caesarean section had significantly lower levels of Th1‐associated chemokines in the blood. The intestinal bacteria play an important role in the establishment of immune tolerance, and the lack of exposure to facultative anaerobic bacteria that occurs during vaginal delivery may increase susceptibility to atopy,^14^, ^38^, ^39^, ^42^, ^43^ and more directly with atopic dermatitis.^44^

In our study, those born by emergency caesarean section and those born by elective caesarean section had similar risks of developing atopic dermatitis. There are few studies that have examined the different atopic dermatitis outcomes when born by emergency or elective caesarean section. In a Danish birth cohort (*n* = 700), emergency versus elective caesarean sections were associated with distinctly different microflora colonization patterns, and it was postulated that this may elicit different health outcomes.^45^ In other types of atopic conditions, including asthma and food allergy, those born by emergency caesarean have demonstrated a slightly higher risk of atopic disease than those born by elective caesarean although the risks were overlapping.^46^, ^47^ Further investigation into the different caesarean section types and the role of microflora in atopic outcomes is warranted.

Similar to our findings, in the study by McKeever et al (*n* = 24,600), children born by forceps or instrumental delivery had an increased risk of atopic dermatitis than those born by uncomplicated vaginal delivery (incidence rate ratio, 1.21; 95% CI, 1.11–1.31).^18^ There are, however, few studies that have investigated this association, we are aware of only one other by Hancox et al (*n* = 1037), which found no association between instrumental vaginal delivery and atopic dermatitis.^48^ The mechanism for the increased risk of atopy associated with forceps or instrumental vaginal delivery is unknown but may be associated with increased fetal stress that alters the programming of Th1 to a Th2 immune response.^18^

Atopic dermatitis is associated with interactions between multiple genes and environmental factors. In our sibling analyses, in children aged under 1 year, an elevated risk of atopic dermatitis was observed in children born by caesarean section. Diepgen et al., in their study on familial aggregation, showed that a stronger correlation of atopic dermatitis exists between siblings than between siblings and parents, suggesting an important role of shared environmental factors.^49^ We observed a higher risk in siblings born by caesarean section, which would suggest that it is an environmental confounder, which is not shared by siblings, but that the effect of the confounder attenuates as the children grow older as reflected by the lack of association in siblings aged ≥1.

Previous studies have also not compared the temporal risks in different pediatric age groups, despite circa 60% of patients manifesting the condition during the first year of life. The course of the condition can be continuous over the life course or be a relapsing–remitting nature with repeated flare‐ups that wane as the child grows.^50^ Whilst evidence of differences between the molecular phenotype of childhood and longstanding adult atopic dermatitis exists,^50^, ^51^ this has not been researched in the different early pediatric age groups. Both pediatric and adult atopic dermatitis show strong T_h_2 and T_h_22 activation^51^; however, markers of Th‐17 associated inflammation (including interleukin [IL]‐17A, IL‐36, IL‐20, P13/Elafin) are markedly raised in children.^50^, ^51^ The diverse immune signatures observed in different pediatric and adult atopic dermatitis age groups may be associated with different antigen‐specific responses that are induced by different triggers at different ages.

### 4.2 Strengths and limitations

This is a population‐based nationwide study that utilized the extensive Swedish medical and administrative registers and is therefore larger than any previous study in children aged ≤5 years.^17^, ^18^, ^19^, ^20^ The wide range of information collected in the registers enabled us to adjust for important covariates. By grouping the mode of delivery into different categories, and including a sibling control analysis, we were able to account for more factors that may influence atopic dermatitis than has been done previously. Further, by including a sibling comparison, we were able to adjust for constant (time‐invariant), but unobserved, parental, familial, and environmental characteristics, which affect all siblings in a sibling relationship in the same way.

We did not adjust for smoking as it does not have a direct effect on the mode of delivery, and instead, its effects are mediated via birth weight and gestational age. Some studies have shown an increased risk of caesarean section in children born to mothers who smoke during pregnancy^52^; however, this finding is not consistent.^53^ In the study by Goudarzi et al (*n* = 3296),^54^ no association was observed between maternal cotinine levels in the third trimester and eczema at ages 1, 2, and 4. Further, maternal cotinine levels were inversely associated with the prevalence of eczema at age 7, which indicates the persistent and long‐term effect of prenatal exposure to cotinine on eczema until age 7.^54^

Our study has some limitations. Some important variables could not be controlled or accounted for and thus we cannot exclude residual confounding,^55^ for example, paternal atopy. Neither could we explore possible mechanisms that may help to explain the association between caesarean section and atopic dermatitis such as breastfeeding. It is known that delivery by caesarean section reduces the likelihood of early initiation and increases the likelihood of early cessation of breastfeeding, and this may further delay the maturation of the gut microbiome.^55^ This study does not represent children with mild disease who may have received over‐the‐counter medication or who had been seen by a general practitioner without being prescribed medication.

## 5 CONCLUSION

In this population‐based cohort study, there was an association between birth by caesarean section and atopic dermatitis. This risk remained when those born by instrumental delivery, emergency, and elective caesarean delivery were further compared with children born by uncomplicated vaginal delivery The observed risks were confounded by genetic and environmental factors in children aged between one and 5 years, but not in the younger children. Further research is needed to determine the mechanisms associated with the risks observed in the different age categories as different periods of onset may signify different subtypes of atopic dermatitis.

## AUTHOR CONTRIBUTIONS

**Mwenya Mubanga:** Conceptualization (equal); formal analysis (lead); methodology (equal); project administration (equal); validation (equal); visualization (lead); writing – original draft (lead); writing – review and editing (equal). **Cecilia Lundholm:** Formal analysis (supporting); methodology (equal); project administration (supporting); supervision (supporting); validation (equal); writing – review and editing (equal). **Elin Rohlin:** Conceptualization (supporting); formal analysis (supporting); writing – original draft (supporting); writing – review and editing (supporting). **Gustaf Rejnö:** Formal analysis (supporting); methodology (supporting); validation (supporting); writing – review and editing (supporting). **Bronwyn Brew:** Formal analysis (supporting); methodology (supporting); validation (supporting); writing – review and editing (supporting). **Catarina Almqvist:** Conceptualization (equal); formal analysis (supporting); funding acquisition (lead); methodology (equal); project administration (lead); resources (lead); supervision (lead); validation (supporting); visualization (supporting); writing – review and editing (equal).

## FUNDING INFORMATION

Financial support was provided by the Swedish Research Council (grant no 2018–02640), the Swedish Heart‐Lung Foundation (grant no 2021041622), and the Stiftelsen Frimurare Barnhuset i Stockholm.

## CONFLICT OF INTEREST

The authors declare no conflict of interest.

### PEER REVIEW

The peer review history for this article is available at https://publons.com/publon/10.1111/pai.13904.

## Supporting information


AppendixS1
