# Supplementary material for: Mode of delivery and offspring atopic dermatitis in a Swedish nationwide study
Source: Pediatr Allergy Immunol. 2023 Jan 11;34(1):e13904. doi: 10.1111/pai.13904 (PMC10107099; doi:10.1111/pai.13904)

**Supplementary Material**

**Mode of delivery and atopic dermatitis in a Swedish nationwide study**

*Mwenya Mubanga,^1^ Cecilia Lundholm,^1^ Elin S Rohlin,^1^ Gustaf Rejnö,^1,2^ Bronwyn K. Brew,^1,3^ and Catarina Almqvist,^1,4^*

**Supplementary text**: Algorithm to define children with atopic dermatitis

**Supplementary Table 1**: Baseline characteristics with extended definition of vaginal delivery and caesarean section.

**Supplementary Figure 1**: Study population

**Supplementary** **Figure 2**: Directed acyclic graph

**Supplementary Figure 3:** Kaplan Meier Survival Estimates

**Algorithm to define children with atopic dermatitis**^53^

Children with atopic dermatitis fulfilled either criterion 1 – 3.

**CRITERIA 1 (ICD-10):**

≥1 hospital contact for:

L20 “atopic dermatitis”

L308C “winter feet”

**CRITERIA 2 (based on ATC):**

≥1 filled prescription of: D11AH “agents for dermatitis: tacrolimus, pimecrolimus” **without any of the exclusion criteria specified below**

**CRITERIA 3 (based on ATC):**

≥ 2 filled prescriptions of: D07 “corticosteroids for topical use” within 12 months **without any of the exclusion criteria specified below**

Without co-occurring hospital contacts and/ or combination of filled prescriptions below (exclusions criteria): Children WITHOUT L20 “atopic dermatitis” with a diagnosis of: L21 “seborrhoeic dermatitis”, L22 “diaper dermatitis” L23 “allergic contact dermatitis”, L24 “irritant contact dermatitis”, L25 “unspecified contact dermatitis”, L26 “exfoliative dermatitis”, L27 “dermatitis due to substances taken internally”, L28 “lichen simplex chronicus and prurigo”, L29 “pruritus”, L30 “other dermatitis” (except L308C), L40 – L45 “papulosquamous disorders”, L53 “other erythematosus conditions”, L55 “sunburn”, L56 “other acute skin changes due to ultraviolet radiation”, L80 “vitiligo”, L90 “atrophic disorders of the skin”, L93 “lupus erythematosus”

OR/AND

**Exclusion medication criteria:**

≥1 filled prescription of either:

D05 “antipsoriasics” or D02AF “salicylates for dermatological use” or D07XB “corticosteroids moderate or potent other combinations “or D07XC “corticosteroids moderate or potent other combinations“ or D07AD01 “corticosteroids (group IV) clobetasol” or D07CD01 “clobetasol and antibiotics”) AND D01 “antifungals” (implies corticosteroid (group IV) use for vaginal fungal infection)

*For prescription of corticosteroid group IV, a filled prescription of group I-III should also be used (as atopic dermatitis is never treated alone with group IV

**Supplementary Table 1: Baseline characteristics with extended definition of vaginal delivery and caesarean section.**

|  | **Total Population** | **Uncomplicated Vaginal Delivery** | **Instrumental Vaginal Delivery** | **Emergency Caesarean Section** | **Elective Caesarean**  **Section** |
| --- | --- | --- | --- | --- | --- |
| **Child Characteristics** |  |  |  |  |  |
| Population |  |  |  |  |  |
| *N (%)* | 1,399,406 (100) | 1,056,323 (75.5) | 94,573 (6.8) | 140,806 (10.0) | 107,704 (7.7) |
| Child’s Age in years |  |  |  |  |  |
| *Mean, (SD)* | 4.3 (1.3) | 4.3 (1.3) | 4.3 (1.4) | 4.3 (1.4) | 4.3 (1.4) |
| Sex |  |  |  |  |  |
| *Male* | 719,609 (51.4) | 533,743 (50.5) | 53,811 (56.9) | 77,304 (54.9) | 54,751 (50.8) |
| Gestational age (weeks) |  |  |  |  |  |
| *≤31* | 12,318 (0.9) | 4,245 (0.4) | 109 (0.1) | 7,325 (5.2) | 639 (0.6) |
| *32-34* | 20,584 (1.5) | 9,404 (0.9) | 662 (0.7) | 8,848 (6.3) | 1,670 (1.6) |
| *35-36* | 47,026 (3.4) | 29,262 (2.8) | 2,441 (2.6) | 11,928 (8.5) | 3,397 (3.2) |
| *37-38* | 265,423 (19.0) | 170,226 (16.1) | 11,618 (12.3) | 26,999 (19.2) | 56,580 (52.5) |
| *39-40* | 710,282 (50.7) | 579,094 (54.8) | 45,308 (47.9) | 43,439 (30.8) | 42,441 (39.4) |
| *≥41* | 343,474 (24.5) | 263,869 (25.0) | 34,418 (36.4) | 42,222 (30.0) | 2,965 (2.8) |
| *Missing* | 299 (<0.0) | 223 (<0.0) | 17 (<0.0) | 47 (<0.0) | 12 (<0.0) |
| Birthweight (grams) |  |  |  |  |  |
| *Mean (SD)* | 3511 (583) | 3543 (529) | 3545 (524) | 3308 (880) | 3443 (594) |
| **Maternal Characteristics** |  |  |  |  |  |
| Maternal age (years) |  |  |  |  |  |
| *Mean (SD)* | 30.4 (5.2) | 30.1 (5.1) | 30.0 (5.1) | 31.2 (5.4) | 32.6 (5.2) |
| Parity |  |  |  |  |  |
| *1* | 605,044 (43.2) | 415,385 (39.2) | 73,840 (78.1) | 84,073 (59.7) | 31,746 (29.5) |
| *2* | 516,968 (36.9) | 416,548 (39.4) | 16,153 (17.1) | 38,287 (27.2) | 45,980 (42.7) |
| *3* | 192,460 (13.8) | 155,497 (14.7) | 3,381 (3.6) | 12,128 (8.6) | 21,454 (19.9) |
| *≥4* | 84,934 (6.1) | 68,893 (6.5) | 1,199 (1.3) | 6,318 (4.5) | 8,524 (7.9) |
| Level of Education |  |  |  |  |  |
| *Compulsory* | 127,826 (9.1) | 99,456 (9.5) | 6,526 (6.9) | 12,857 (9.1) | 8,987 (8.3) |
| *Secondary* | 494,097 (35.3) | 375,127 (35.5) | 31,074 (32.9) | 50,541 (35.9) | 37,355 (34.7) |
| *Tertiary* | 766,126 (54.8) | 572,840 (54.2) | 56,324 (59.6) | 76,265 (54.2) | 60,697 (56.4) |
| *Missing* | 11,357 (0.8) | 8,900 (0.8) | 649 (0.7) | 1,143 (0.8) | 665 (0.6) |

Children born Jan 2006 to Dec 2018

N=1,400,713

Excluded:

21 children excluded for missing maternal or child related data;

1,286 Missing mode of delivery

Vaginal delivery

Migrated = 1,931 (0.2%)

Died = 2,482 (0.2%)

Atopic dermatitis =197,871 (17.2%)

Eligible children

N=1,399,406

Died-4286

Emigrated-2254

Caesarean section delivery

Migrated = 323 (0.1%)

Died = 1,804 (0.7%)

Atopic dermatitis = 45,422 (18.3%)

**Supplementary Figure 1: Study population**

**Supplementary** **Figure 2**: Directed acyclic graph


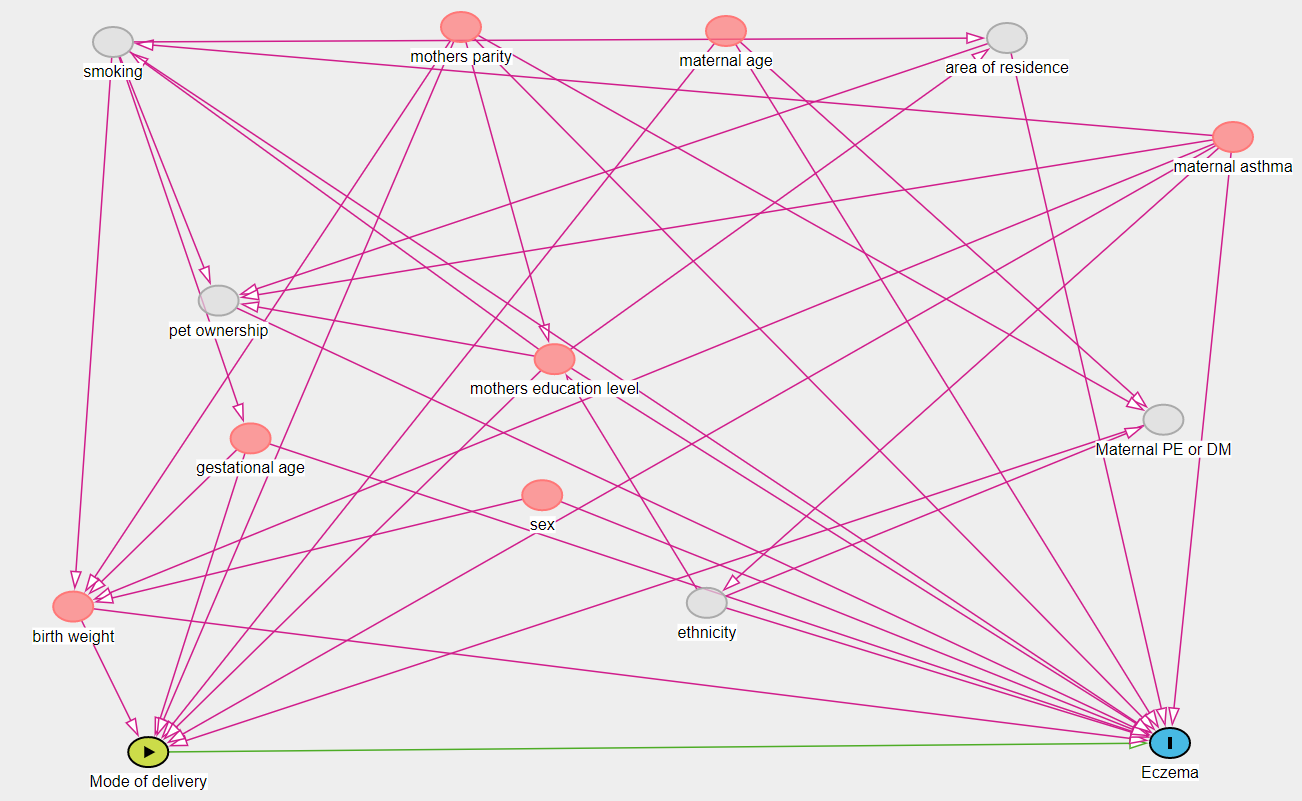


| LEGEND | |
| --- | --- |
| 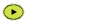 | Exposure |
| 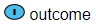 | Outcome |
| 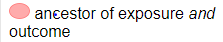 | Confounder included in final model |
| 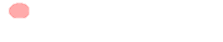 | Variables not included in final model |

**Supplementary Figure 3. Kaplan Meier Survival estimates**


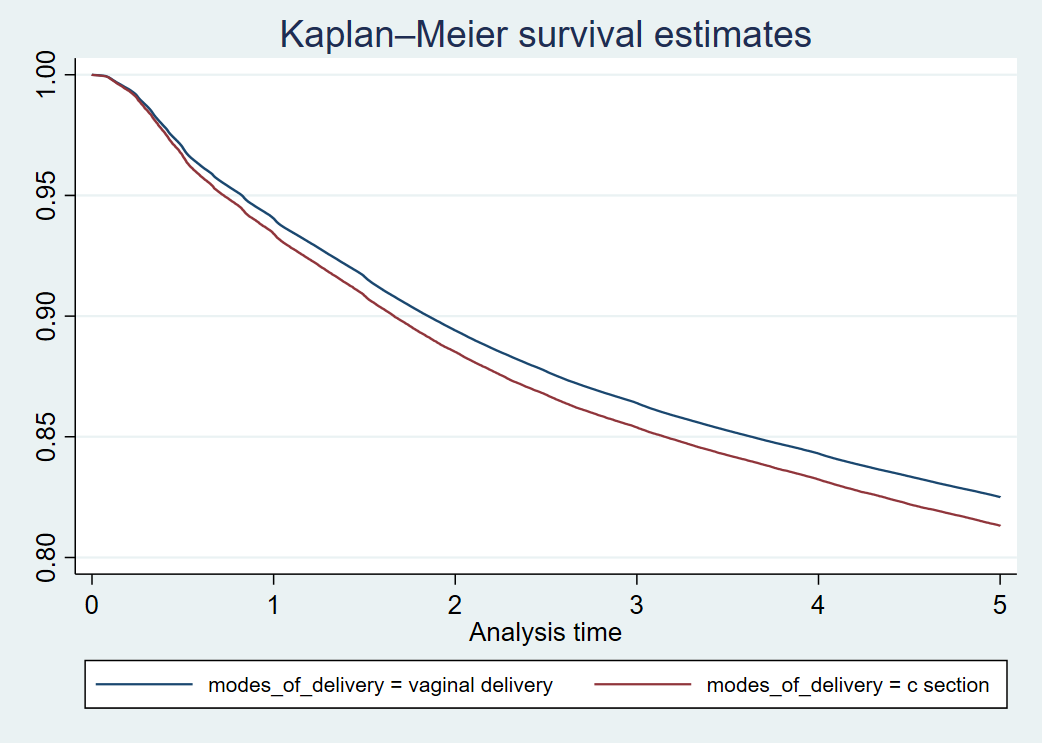

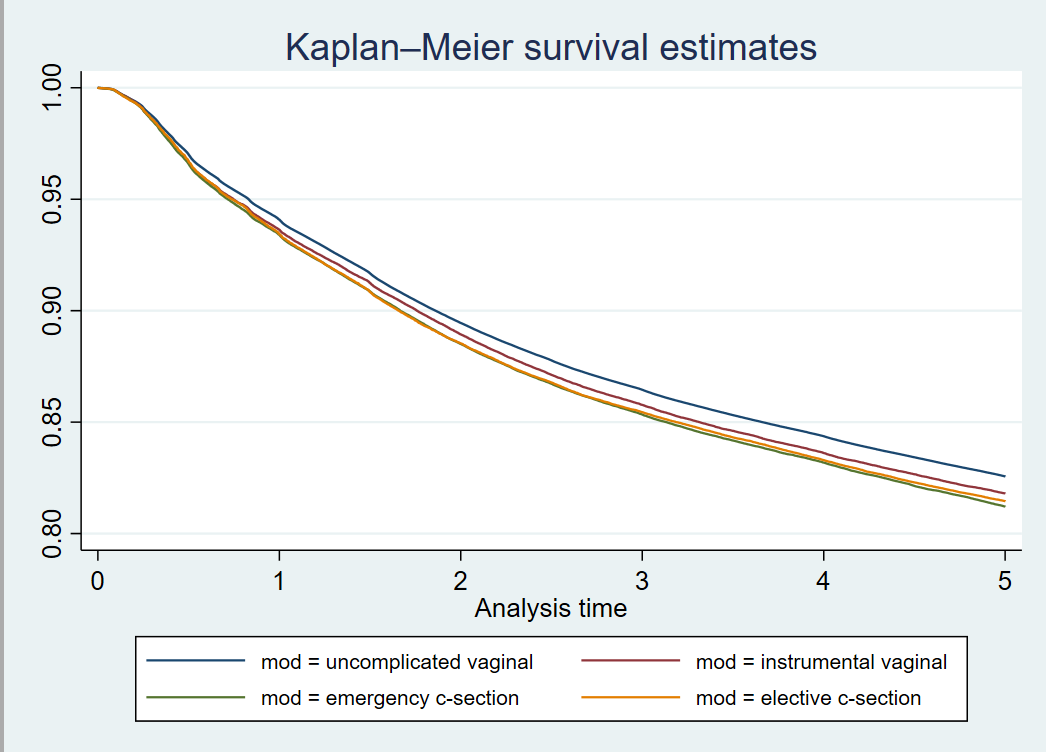

Supplement: Supplementary file 1 — AppendixS1 [file PAI-34-0-s001.docx]
